# Utility of Pelvic Ultrasound with Negative Computed Tomography in Adult Females

**DOI:** 10.5811/westjem.48919

**Published:** 2026-05-19

**Authors:** Mary Rometti, Amanda Esposito, Michael Mirza, Sara Heinert, Christopher Bryczkowski

**Affiliations:** Rutgers Health Robert Wood Johnson Medical School, Department of Emergency Medicine, New Brunswick, New Jersey

## Abstract

**Introduction:**

Emergency physicians must consider ovarian torsion in all biologically female patients who present to the emergency department (ED) with abdominal pain. A misdiagnosis can result in detrimental outcomes, such as loss of an ovary. Female patients who present to the ED for evaluation of their abdominal pain may have a computed tomography (CT) or pelvic ultrasound performed to further evaluate for concerning pathology. Our objective in this study was to describe the occurrence of critical or emergent findings on pelvic ultrasound not identified on a concurrent or previously performed CT of the abdomen and pelvis.

**Methods:**

We conducted a retrospective chart review of ED visits from January 1–December 31, 2021 at a large, suburban, academic medical center. Eligible patients were adult females (≥ 21 years of age) who had a CT of the abdomen and pelvis and pelvic ultrasound, with the CT ordered either before or simultaneously with the pelvic ultrasound. We excluded from the study CTs with abdominal or pelvic pathology. The primary outcome measure was to determine the occurrence of pelvic ultrasounds without acute pathology with a negative CT. The secondary outcome measure was to identify non-emergent findings on pelvic ultrasound with a negative CT.

**Results:**

Of 281 eligible ED visits, 172 patients (61.2%) had gynecologic pathology on CT and 60 patients (21.4%) had other pathology on CT. The mean age was 43.3 years (SD 16.4). Forty-nine patients (17.4%) had unremarkable CT results. Of those, 39 patients (79.6%; 95% CI, 65.7–89.8%) had a normal ultrasound; three patients (6.1%; 95% CI,1.3–16.9%) had an ovarian cyst; six patients (12.2%; 95% CI, 4.6–24.8%) had other non-emergent results; and one patient (2.0%; 95% CI, 0.05–10.9%) had a 10- by 11-mm ovarian mass on ultrasound. In patients with unremarkable CTs, 48 (98.0%; 95% CI, 89.1–99.9%) also had normal or not clinically significant ultrasound results. No definite ovarian torsions were diagnosed on ultrasound after a negative CT.

**Conclusion:**

Obtaining a pelvic ultrasound following an unremarkable CT of the abdomen and pelvis may not produce additional clinically relevant results in an emergency setting.

## INTRODUCTION

Abdominal pain is a common presentation to the emergency department (ED) and includes a broad differential.^1^–^3^ Ovarian torsion is the fifth most common acute gynecologic complaint for biological females.^1^ While ovarian torsion is still considered a rare diagnosis, emergency physicians must consider it in the differential for female patients with abdominal pain given that misdiagnosis can result in ovarian necrosis or infertility.^1^–^4^

Clinical presentations of a patient with ovarian torsion may vary, which further contributes to the challenge of diagnosing torsion.^1^,^5^,^6^ Ovarian torsion most commonly occurs in reproductive-age females, with an average age of onset of about 30 years.^1^,^4^ About half of patients with torsion present with abrupt onset of pelvic pain with radiation to the flank or groin area.^1^ The abdominal or pelvic pain associated with ovarian torsion is primarily from vascular occlusion leading to ischemia.^1^ Patients may present with nausea, vomiting, intermittent or constant pain, fever, tachycardia, or hypertension.^1^ Risk factors for ovarian torsion include prior torsion, ovarian hyperstimulation interventions, polycystic ovarian syndrome, adnexal masses or cysts, pregnancy, and prior tubal ligations.^3^,^4^,^7^ The risk of ovarian torsion increases when cysts are greater than 5 cm in diameter, as increased size may lead to the ovary rotating along its axis, impeding blood flow.^8^

Up to 65% of patients with pelvic or abdominal pain in the ED will have imaging performed in the emergency setting.^3^ To evaluate ovarian torsion, transvaginal ultrasound with Doppler is the initial recommended test.^1^–^4^ When presenting to the ED with lower abdominal or pelvic pain, a patient will often initially have computed tomography (CT) performed to evaluate for causes of lower abdominal pain.^1^ At times, both an ultrasound and a CT may be ordered.^9^–^11^ Although additional studies may be necessary, some findings on CT may aid in either diagnosing ovarian pathology related to torsion or lowering the diagnostic possibility of ovarian torsion. Using these CT findings may decrease patient discomfort related to transvaginal ultrasound studies.^1^,^6^ An unremarkable abdominal/pelvis CT greatly reduces the likelihood of ovarian torsion.^1^ An ultrasound performed immediately after a negative CT likely has little diagnostic function.^3^ One study found that CT and ultrasound had comparable diagnostic performances when evaluating for ovarian torsion when confirmed by surgery.^3^,^6^

Our objective in this study was to determine the occurrence of critical or emergent pelvic ultrasound findings not identified on concurrent or previously performed CT of the abdomen and pelvis. If the pelvic ultrasound is unlikely to reveal clinically significant pathology, a subsequent ultrasound may not be indicated, thereby decreasing the length of time spent in the ED and the cost of patient care.

## METHODS

We performed a retrospective chart review of ED presentations for abdominal pain from January 1–December 31, 2021. Charts were extracted from AllScripts Sunrise Clinical Manager (Altera Digital Health, Inc, Niagara Falls, NY) and the ED information management system for patients who met the following criteria: females ≥ 21 years of age presenting with abdominal pain, not pregnant, who had both a CT of the abdomen and pelvis and pelvic ultrasound performed, with the CT ordered either prior to or simultaneously with the ultrasound. We also extracted return visit data for the seven-day period following the patient’s day of initial presentation, extending to January 7, 2022. The primary outcome measure was to determine the occurrence of unremarkable pelvic ultrasounds with a negative CT of the abdomen and pelvis. The secondary outcome measure was to identify clinically important or unimportant nonemergent findings on pelvic ultrasound with a negative CT.

**Population Health Research Capsule**

What do we already know about this issue?

*While a transvaginal ultrasound with Doppler is historically the recommended test to assess for ovarian torsion, CT is often also performed*.

What was the research question?

*After an unremarkable CT of the pelvis and abdomen, does a pelvic ultrasound provide additional clinically useful information?*

What was the major finding of the study?

*Of 49 patients with normal CTs, 48 (98.0%; 95% CI, 89.1–99.9) had clinically insignificant ultrasound results*.

How does this improve population health?

*Two different imaging modalities may not be required to rule out ovarian torsion. If validated with a larger sample size, patients may not need to undergo additional imaging*.

We initially obtained data using an administrative data query with additional manual data extracted from the charts by three study authors. Data were stored using REDCap tools (Research Electronic Data Capture, Vanderbilt University, Nashville, TN) hosted at Rutgers Health, and were further analyzed in Excel (Microsoft Corporation, Redmond, WA) and Stata (StataCorp, LLC, College Station, TX). As outlined by Worster et al, we adhered to the following guidelines for retrospective chart review: abstractor training; case selection criteria; variable definition; abstraction forms; performance monitored; medical record identified; and institutional review board approval.^12^

## RESULTS

**Figure 1:**
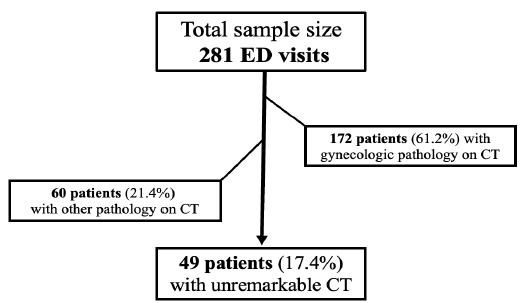
Sample size with subsequent exclusions for emergency department presentations of female patients with computed tomography of the abdomen and pelvis ordered initially or simultaneously with pelvic ultrasound. *CT*, computed tomography of the abdomen and pelvis; *ED*, emergency department.

Of 281 patients for whom a CT of the abdomen and pelvic and pelvis ultrasound was ordered, the mean age was 43.3 years (SD 16.4); 9% identified as Asian, 15% as Black, 37% as White, and 32% identified as having Hispanic ethnicity. Spanish was the primary language for 57 (20%) patients. A total of 172 patients (61.2%) had gynecologic pathology seen on CT, and 60 patients (21.4%) had other pathology on CT, while 49 patients (17.4%) had unremarkable CT results. (Figure 1).

Of the pelvic ultrasound results examined for the 49 patients with unremarkable CTs, 39 were normal (79.6%; 95% CI, 65.7–89.8%). Ovarian cysts were found in three patients (6.1%; 95% CI, 1.3–16.9%), all of whom had normal duplex scans. The largest cyst of the three patients measured 2.3 cm. Non-emergent results of fibroids, nabothian cyst, ovarian follicle, hydrosalpinx, and thickened endometrium were identified in six patients (12.2%; 95% CI, 4.6–24.8%). Of these six, one had a renal transplant ultrasound performed in the ED followed by pelvic ultrasound a few days later that showed questionable torsion, later identified as likely the transplanted kidney. Finally, an ovarian mass was found in one patient (2.0%; 95% CI, 0.05–10.9%), which was characterized as 10 mm by 11 mm and non-specific (unable to exclude neoplasm). This patient had a normal duplex on ultrasound. Results are summarized in Table 1. Overall, 48 patients (98.0%; 95% CI, 89.1–99.9%) had clinically insignificant US results. No ovarian torsions were diagnosed on ultrasound after a negative CT.

## DISCUSSION

While ovarian torsion is a rare diagnosis, it is considered a gynecologic emergency. Efficiently diagnosing or ruling out ovarian torsion is vital. While CT is generally not considered the gold standard for diagnosing ovarian torsion, it may be the preferred imaging modality in the ED.^7^ While a CT of the abdomen and pelvis cannot detect dynamic blood flow like an ultrasound, it can depict other findings or risk factors that could signify an ovarian torsion. Ovarian torsion is typically associated with pelvic pathology, such as an ovarian mass, which can result in twisting along the ligaments, compressing vasculature.^4^

Computed tomography of the abdomen and pelvis with intravenous contrast has been shown to have high sensitivity and specificity when evaluating for possible ovarian torsion.^4^ Specific CT findings for ovarian torsion include decreased ovarian contrast enhancement, ovarian follicles that are peripherally displaced, enlarged ovary with a follicular stroma, and thickened fallopian tube with a beak-like appearance.^4^ Other CT findings that could indicate ovarian torsion include fat-stranding near the ovary, adnexal wall thickening, pelvic free fluid, enlarged ovaries, ovarian masses, shifting of an ovary closer to the uterus, or uterine displacement closer to a torsed ovary.^4^,^11^ If a CT does not show evidence of findings suggestive of ovarian torsion, the sensitivity for ruling out ovarian torsion by CT is close to 100%.^4^ To our knowledge, there is no definitive published literature defining the lower size limit of an ovarian cyst to be detectable on CT. If CT could reliably show there is no ovarian cyst, then the need for an ovarian ultrasound would be further reduced. While CT has been used to aid in characterizing benign vs malignant ovarian cysts by examining fluid attenuation,^13^ additional studies are needed to define CT ovarian cyst size sensitivity.

In one study, the retrospective review of initial CTs for surgically confirmed ovarian torsion diagnoses revealed that the CTs had at least one abnormal finding that could have been associated with ovarian torsion.^7^ Patients with ovarian torsion are unlikely to have a normal CT.^11^ Given that ovarian torsion is often the result of enlargement of the ovaries or asymmetrical ovaries, the CT should show evidence of this or secondary findings. Even though Viers et al observed that in 34% (32/93) of cases, transvaginal ultrasound may have helped to more definitively exclude ovarian torsion, they also found that transvaginal ultrasound performed after CT revealed a new or different diagnosis in < 1% of their patient population.^9^ Furthermore, performing a CT after ultrasound led to a new or different diagnosis in about 26% of cases.^9^ Pelvic ultrasound after normal findings on CT could aid in diagnosing uterine or endometrial pathology but otherwise has no emergent clinical benefit.^9^,^10^ Performing ultrasound after a normal CT with normal pelvic organs is likely not to be beneficial or yield additional diagnostic information required in the emergency care setting.^10^,^11^ Our results are consistent with these prior studies, demonstrating that those patients with unimportant CT findings will not benefit from an emergent pelvic ultrasound to rule out torsion. Future studies with larger sample sizes are necessary to confirm these conclusions.

## LIMITATIONS

There are some limitations within this study. First, after patients with non-ovarian pathology on CT were removed, the resulting sample size was small. A larger sample could have increased the reliability of the data. Second, there is the potential for confounders, such as whether the CT and ultrasound were ordered simultaneously vs the CT first. Third, the data were abstracted from charts by study authors who were not blinded to the study hypothesis; however, this is unlikely to have significantly contributed as the information collected was mainly fact-based.

## CONCLUSION

Among the many potential intra-abdominal pathologies seen in the ED, emergency physicians must assess and rule out significant life- or organ-threatening diagnoses in a timely and efficient manner. Traditionally, a pelvic ultrasound is used to assess for ovarian torsion; however, this study suggests that it may not be emergently necessary if the patient already had an unremarkable CT of the abdomen and pelvis.

## Figures and Tables

**Table 1:** Breakdown of findings on pelvic ultrasound for patients who had unremarkable computed tomography of the abdomen and pelvis.

Clinical importance	Findings on ultrasound	Number of patients (%, 95% CI)
Unimportant		
	Normal ultrasound	39 (79.6%, 65.7–89.8%)
	Ovarian cyst	3 (6.1%, 1.3–16.9%)
	Non-emergent results	6 (12.2%, 4.6–24.8%)
Important		
	Ovarian mass	1 (2.0%, 0.05–10.9%)
